# O-31 - REDUCING ANTIBIOTIC ELIGIBILITY FROM 21 TO 14 DAYS AFTER SYMPTOM ONSET FOR CASES AND CONTACTS OF PERTUSSIS IN ENGLAND: NO EVIDENCE OF INCREASED HOUSEHOLD CLUSTERING

**DOI:** 10.1093/pubmed/fdag046.027

**Published:** 2026-07-09

**Authors:** Ms Eleanor Clarke, Dr Stephanie Newton, Dr Amoolya Vusirikala, Dr Helen Campbell, Dr Ellen Heinsbroek, Dr Karthik Paranthaman

**Affiliations:** UK Health Security Agency; UK Health Security Agency; UK Health Security Agency; UK Health Security Agency; UK Health Security Agency; UK Health Security Agency

## Abstract

**Background:**

High pertussis activity in England necessitated urgent revisions to public health (PH) recommendations in June 2024. An important change was the reduction in timeframe for advising antibiotic therapy for cases without high-risk contacts and eligible contacts from 21 to 14 days from symptom onset.

**Objectives:**

We investigated whether the 2024 recommendations were associated with increased household transmission compared to earlier periods.

**Methods:**

Data on all notified cases in England between January 2018 and February 2026 was extracted from the Case and Incident Management System (CIMS). Residential address was linked to a Unique Property Reference Number (UPRN), a national identifier that uniquely distinguishes each UK property. Clusters were defined as two or more cases with symptom onset within 90 days of another case at the same UPRN. Clusters were categorised into four time periods according to the level of pertussis activity (high or endemic) and prevailing PH recommendations (21-day or 14-day). We compared the proportion of clustered cases across these periods, and examined differences in age, sex and deprivation distribution across the same periods.

**Results:**

A total of 3,601 clusters with 55,112 cases (median age 29 years) were included in the analysis, of which 14.4% were clustered. During high transmission, 17.5% of cases were clustered in the 21-day period and 12.2% in the 14-day period (p<0.001). The corresponding proportions during endemic transmission were 15.6% and 8.2% (p<0.001). Age, sex and deprivation distribution were similar in all periods.

**Conclusions:**

Reducing the antibiotic eligibility timeframe from 21 to 14 days was not associated with increased household clustering. Although limited by absence of detailed individual-level data, these findings offer cautious reassurance that the 2024 guidance did not have an adverse impact on household transmission. Further studies using enhanced epidemiological data are needed to confirm these findings and inform refinements to PH management guidance.

